# Editorial: The role of glia in plasticity and behavior

**DOI:** 10.3389/fncel.2015.00356

**Published:** 2015-09-09

**Authors:** Tycho M. Hoogland, Vladimir Parpura

**Affiliations:** ^1^Netherlands Institute for NeuroscienceAmsterdam, Netherlands; ^2^Department of Neuroscience, Erasmus MCRotterdam, Netherlands; ^3^Atomic Force Microscopy and Nanotechnology Laboratories, Department of Neurobiology, Center for Glial Biology in Medicine, Civitan International Research Center, Evelyn F. McKnight Brain Institute, University of Alabama at BirminghamBirmingham, AL, USA

**Keywords:** astrocytes, glia, behavior, plasticity, transgenic mouse models

New genetic tools have not only advanced our understanding of how neurons contribute to plasticity and behavior, but also unveiled glial cells as active participants in circuit function as highlighted in this Research Topic. Glia are found in the majority of animals with a nervous system and are essential for feeding and learning behavior in a “simple” organism such as *C. elegans* (Stout et al., 2014), which has a nervous system invariantly comprising 302 neurons. Glial cells signaling via gap junctions may even have evolved convergently during evolution suggesting that spreading activity via such cellular networks is a fundamental process (Stout et al., 2014).

In more complex mammalian brains, glia respond to a variety of neurotransmitters and could be instrumental for gauging metabolic demand in times of increased vigilance -a behavioral state mediated by adrenoreceptors (Ding et al., 2013)- across brain regions (Paukert et al., 2014; De Zeeuw and Hoogland, 2015). Pankratov and Lalo (2015) present data suggesting that the activation of astroglial α1-adrenergic receptors and subsequent exocytotic release of ATP from these glial cells are important for the induction of long-term potentiation in neocortical neurons. Thus, the behavioral state of the animal, vigilance in the case of adrenergic modulation, has a big impact on the learning capacity of the brain and may rely on gliotransmission.

The role of gliotransmission is still contentious as a recent study has shown that a commonly used transgenic mouse (dnSNARE) which has been utilized to block exclusively gliotransmission (Pascual et al., 2005; Lalo et al., 2014), could lead to ectopic expression (Fujita et al., 2014); discussed in Xie et al. (2015). Bearing this in mind, Hahn et al. (2015) utilized co-cultures of neurons and astrocytes, an approach instrumental in studying astrocyte-to-neuron signaling (Parpura et al., 1994), to demonstrate that astrocytes can increase the activity of NMDA-receptor dependent synaptic transmission at the postsynaptic subunit (N2B)-specific level. Thus, astrocytes not only sense neuromodulatory state, or local circuit activity, but also influence the circuits in which they are embedded, as reviewed by Perea et al. (2014a).

Could astrocytes themselves undergo plasticity? Sibille et al. (2015) addressed this question with dual recordings of hippocampal astrocyte Ca^2+^ signaling and synaptic transmission at Schaffer collateral pathway (SC)-CA1 pyramidal neuron (excitatory) synapses. During repetitive or tetanic stimulation of SC, astrocytes showed short-term depression of cytosolic Ca^2+^ signals associated with a simultaneous short-term potentiation at SC-CA1 synapses. Moreover, chelation of Ca^2+^ in astrocytes resulted in enhanced synaptic transmission and short-term plasticity at SC-CA1 synapses. This finding adds to a growing body of evidence that astrocytes are critical for certain types of synaptic plasticity (Min and Nevian, 2012).

Although evidence is accumulating that glial cells regulate the excitability of neural circuits and could therefore contribute to behavior -at least in certain animals- there are also other controversies that need to be resolved. One of them is the role of Ca^2+^ signaling in astrocytes that has been considered an essential feature of astrocyte function and plasticity (Nimmerjahn et al., 2009; Sibille et al., 2015).

Following up on work that proposed that Gq protein-coupled receptor Ca^2+^ mobilization in astrocytes does not affect neuronal synaptic transmission (Agulhon et al., 2010) or synaptic plasticity in hippocampal astrocytes, Petravicz et al. (2014) recently demonstrated in an inositol 1,4,5, trisphosphate receptor type 2 (otherwise providing for Ca^2+^ dynamics in astrocytes) conditional knockout mouse line that a battery of behavioral assessments (Morris water maze, elevated plus-maze, rotarod, open field activity, and acoustic startle response tests) failed to reveal any behavioral deficits. The above studies are reviewed by Xie et al. (2015), who also summarize the toolbox of currently available genetic approaches that enable the study of astrocytes *in vivo*.

Although optogenetics has proven to be clearly advantageous for studying neuronal correlates of behavior and functional connectivity in the brain (Friedman et al., 2015; Pala and Petersen, 2015), and it has been applied to selectively stimulate astrocytes (Gourine et al., 2010; Sasaki et al., 2012; Perea et al., 2014b; Natsubori et al., 2015), there are some caveats to its use in general and astrocytes in particular. These include the non-selectivity of, e.g., channelrhodopsin (ChR2) channels for cations leading to intracellular acidification (Beppu et al., 2014; also see Natsubori et al., 2015), as well as large Ca^2+^ increases that could bypass downstream signaling cascades, and which result in non-specific modulation of astrocyte function (Wang et al., 2013). Xie et al. (2015) suggest that Designer Receptors Exclusively Activated by Designer Drugs (DREADD) provide a more precise method to reversibly control astrocyte activity allowing the disentanglement of the signaling pathways that contribute to glial modulation of circuit activity.

What are the current obstacles that need to be overcome to advance the field? Jahn et al. (2015), Xie et al. (2015), and Natsubori et al. (2015) all note that there is a need for better genetic models to study astrocyte function. Such models would allow inducible knockout, expression of proteins involved in astrocyte signaling at appropriate levels, and would also make use of more specific promotors that could target various types of glial cells in different brain areas. Furthermore, Jahn et al. (2015) stress the importance of considering the lifetime of proteins. Namely, recent data obtained from Bergmann glia revealed that knockout of subtypes of AMPA receptors resulted in process retraction, which fully manifested itself only after several weeks, a time-frame matching protein degradation of these channels (Saab et al., 2012).

The goal of this Research Topic has been to summarize our most recent understanding of glial cells in the regulation of plasticity of neural circuits. It is abundantly clear that genetic approaches have been instrumental in elucidating the role of glia in plasticity and behavior and that outstanding issues are likely to be resolved in the near future with novel genetic tools. Thus, exciting times lie ahead for the study of astrocytes in the dish and the behaving brain.

## Conflict of interest statement

The authors declare that the research was conducted in the absence of any commercial or financial relationships that could be construed as a potential conflict of interest.
